# Prospective exploratory study to assess the safety and efficacy of aflibercept in cystoid macular oedema associated with retinitis pigmentosa

**DOI:** 10.1136/bjophthalmol-2019-315152

**Published:** 2020-09-01

**Authors:** Stacey A Strong, Tunde Peto, Catey Bunce, Wen Xing, Michalis Georgiou, Simona Degli Esposti, Angelos Kalitzeos, Andrew Webster, Michel Michaelides

**Affiliations:** 1 UCL Institute of Ophthalmology, University College London, London, UK; 2 Moorfields Eye Hospital, London, UK; 3 Queen’s University Belfast, Belfast, UK; 4 Primary Care and Public Health Sciences, Kings College London, London, UK

**Keywords:** retina, macula

## Abstract

**Aims:**

To report the safety and efficacy of intravitreal aflibercept (Eylea) (ivA) for retinitis pigmentosa-associated cystoid macular oedema (RP-CMO) at 12 months via mean central macular thickness (CMT) and reported adverse events.

**Methods:**

A prospective, exploratory, phase II, non-randomised, single-centre, open-label, 1-arm clinical trial involving 30 eyes of 30 patients. Serial ivA was given via loading dose (three injections) followed by treat and extend protocol over 12 months.

**Results:**

Twenty-nine out of 30 (96.7%) patients completed 12 months of follow-up. A total of four to 11 injections per patient were given over the 12 month study. No statistically significant reduction of CMT or visual acuity (VA) improvement was demonstrated in the group overall. Eleven out of 29 (37.9%) participants were considered as ‘responders’, demonstrating at least an 11% reduction of CMT at 12 months on spectral domain optical coherence tomography compared with baseline. A reduction of CMT by mean (SD) 28.1% (12.9 %) was observed in responders at 12 months, however, no statistically significant corresponding improvement in best corrected VA was seen. Baseline characteristics were similar between responder and non-responder groups. No clinically significant adverse events were deemed secondary to ivA.

**Conclusion:**

This first prospective exploratory study demonstrates both the safety and acceptability of serial ivA in patients with RP-CMO, effective at reducing CMT in 37.9% of patients. All patients demonstrating anatomical response did so after their first injection. Longer duration of CMO did not negatively affect response to anti-VEGF. Further study in a larger cohort of patients with shorter CMO duration would be valuable to better establish the utility of VEGF blockade in RP-CMO.

**Trial registration numbers:**

EudraCT (2015-003723-65); ClinicalTrials.gov (NCT02661711).

## Introduction

Inherited retinal disease is the second most common cause of visual loss in childhood and the most common cause of visual loss in the working age population.^1^ Retinitis pigmentosa-associated cystoid macular oedema (RP-CMO) is a known complication of retinitis pigmentosa (RP), reported to occur in 10%–50% of patients with RP across their lifetime.^2–5^ One of the most commonly reported ocular symptoms of RP is relentless and progressive concentric peripheral visual field loss for which there is currently no cure. Complications of RP such as cataract and RP-CMO interfere with central vision and are thereby particularly debilitating, making effective treatments for RP-CMO highly valuable.

Several mechanisms have been proposed to explain RP-CMO, however, no single aetiology has been definitively established.^6^ These include: (i) breakdown of the blood-retinal barrier,^7 8^ (ii) failure (or dysfunction) of the retinal pigment epithelium (RPE) pump mechanism,^9^ (iii) Müller cell oedema and dysfunction,^10^ (iv) anti-retinal antibodies^11^ and (v) vitreous traction.^12 13^ Treatment approaches for RP-CMO have included: laser therapy, topical carbonic anhydrase inhibitors (CAIs), oral CAIs, periocular and intravitreal steroids, and intravitreal anti-vascular endothelial growth factor (anti-VEGF) agents.^6^ The vast majority of the published literature is retrospective, however, involving only small numbers of participants with short duration of follow-up. RP-CMO has been associated with younger age but not with gender.^14^ RP-CMO is most prevalent in patients with autosomal dominant (AD) inheritance (71.4% with CMO in at least one eye), followed by autosomal recessive (AR)/sporadic inheritance (58.9%) and x-linked (XL) inheritance (12.5%).^14^ Patients with epiretinal membrane (ERM) and cataract/pseudophakia are less likely to develop CMO.^14^

The current mainstay of treatment for RP-CMO is topical/oral CAIs, however, there is no level 1 evidence supporting their use and studies have demonstrated highly variable efficacy. Liew *et al* carried out a 12 month retrospective review of 81 patients with RP-CMO at Moorfields Eye Hospital, UK treated with topical dorzolamide (64 patients, 125 eyes) or oral acetazolamide (17 patients, 32 eyes).^15^ Forty per cent of eyes (53.1% of patients) following treatment with topical dorzolamide and 28.1% of eyes (41.2% of patients) following treatment with oral acetazolamide demonstrated response (defined as a reduction of central macular thickness (CMT) on optical coherence tomography (OCT) of at least 11% between visits).^15^ A cross-sectional study performed on this same cohort of patients (n=81) identified older age, earlier age of onset of symptoms, and thicker CMT to be associated with lower visual acuity (VA). Gender and inheritance pattern were not found to be associated with VA.^16^

Several publications have observed a variable effect of anti-VEGFs in RP-CMO, including: pegaptanib sodium (Macugen, (OSI) Eyetech Pharmaceuticals/Pfizer, New York, New York, USA),^17^ bevacizumab (Avastin, Genentech/Roche, South San Francisco, California, USA),^18 19^ ranibizumab (Lucentis; Genentech, South San Francisco, California, USA)^20 21^ and aflibercept (Eylea; Regeneron Pharmaceuticals, Tarrytown, New York, New York, USA, and Bayer Healthcare Pharmaceuticals, Berlin, Germany).^21 22^ The largest study to-date by Artunay *et al* enrolled 30 eyes of 30 patients with RP-CMO refractory to treatment with oral acetazolamide for at least 6 months.^20^ Fifteen eyes of 15 patients were treated with a single intravitreal injection of ranibizumab (ivR). Fifteen eyes of 15 patients that declined ivR were used as a control group. Thirteen out of 15 eyes (87%) in the treatment group demonstrated significant reduction of CMO at 6 months post-injection, although the definition of ‘significant reduction’ is not stated in the paper. No statistically significant difference in VA was demonstrated in this cohort as a whole, or in subgroup analysis of responders. Moustafa and Moschos published a case report demonstrating improvement of CMT and VA following a single unilateral intravitreal injection of aflibercept (ivA) in a 52-year-old with RP-CMO. At baseline, vision was 3/10. One month post-injection, vision improved to 4/10 and CMO resolved. Documented visual improvement was maintained at both 2-month and 6-month reviews.^22^ Our group subsequently published a case report regarding a 38-year-old patient with a 3-year history of bilateral RP-CMO. Previous treatment had been with topical 2% dorzolamide, oral acetazolamide, and ivR, which had demonstrated only minimal reduction of CMO. He had a good structural response to bilateral doses of ivA. He subsequently received serial ivR with further reduction of CMT observed. VA remained stable throughout.^21^

Given the aforementioned lack of high quality evidence for use of therapeutic options for RP-CMO, we designed a phase II exploratory prospective study to assess the safety and efficacy of ivA in a well-characterised cohort of patients with RP-CMO in order to help provide evidence towards this unmet medical need.

## Methods

Written informed consent was obtained from all patients. The study was undertaken at Moorfields Eye Hospital NHS Foundation Trust, UK. The consort flow diagram illustrating flow of patients throughout the study can be found in online supplementary figure 1.

10.1136/bjophthalmol-2019-315152.supp1 Supplementary data

### Identification of suitable patients for the trial

An electronic search was performed to identify all patients seen at Moorfields Eye Hospital NHS Foundation Trust, UK, between 1st December 2012 and 30th November 2015 with the phrases ‘retinitis pigmentosa’ and ‘cystoid macular oedema’ appearing in their electronic patient records. This initial search identified 295 patients; however, after review of each electronic patient record and latest Spectral domain OCT (SDOCT) imaging, 165 patients were excluded from the study for the following reasons: no/minimal CMO (111), visually significant ERM (17), VA too poor (24), VA too good (4), macular hole (2), visually significant cataract (2), under 16 years of age (4) and pregnant (1). Please refer to online supplementary information 1 for a list of inclusion/exclusion criteria.

10.1136/bjophthalmol-2019-315152.supp2 Supplementary data

A total of 130 patients were therefore found to be potentially suitable participants. Patients were contacted by the dedicated trial fellow (SAS) at their routine medical retina clinic or by telephone/letter. The aims, methods, anticipated benefits and potential hazards of the study were explained to each patient and a patient information sheet provided. Patients were given a minimum of 24 hours to consider whether they wished to attend a baseline evaluation/screening visit. Of these patients: 18 could not be contacted/did not reply, 1 was deceased, 32 wished to be considered for the study, and 79 declined to participate for reasons including: did not want intravitreal injections (n=42), happy with their current treatment and/or vision (n=22), or unable to commit to the study visits (due to distance from the hospital or concerns about the impact it would have on their job) (n=15).

Out of 32 patients who wished to be considered for the study, 15 patients were being treated with a topical CAI (dorzolamide or brinzolamide) and five patients were being treated with an oral CAI (acetazolamide) at time of contact. Patients were requested to stop using CAIs for at least 1 month in the study eye if being used topically, or at least 3 months if orally, before their screening appointment was made. Ten patients were not using any treatment. Two patients had no CMO at screening so were excluded from the trial.

### Recruitment period

All 30 patients were recruited over a 6-month period.

### Baseline evaluation/screening visit

At the screening appointment, each patient had the opportunity to ask further questions before written informed consent was taken. Baseline tests of visual function can be found in online supplementary information 2.

10.1136/bjophthalmol-2019-315152.supp3 Supplementary data

If a patient was deemed eligible to enter the trial, intra-ocular pressure (IOP) was measured using Goldmann tonometry and their first ivA was given that day (‘Visit 1’). The IOP was re-checked 30 min after ivA, and appropriate treatment commenced if IOP was increased (≥30 mm Hg).

### Randomisation

The study consisted of only one-arm and all trial patients received the active drug, aflibercept via intravitreal injection.

### Follow-up visits

At each follow-up visit, patients had their vital signs checked and a medication review performed. Tests of visual function carried out at every visit, included: best corrected VA (BCVA), colour vision, contrast sensitivity and SDOCT. In addition, microperimetry and fundus autofluorescence were undertaken at the 6-month and 12-month (exit) visits. IvA was administered every 4 weeks for the first 3 months (loading phase), followed by a treat and extend protocol up to 12 months. Extension from monthly to 6, 8, 10 and 12 week follow-up occurred when there was no reduction in macular oedema compared with the previous visit. Please refer to online supplementary table 1 for a schedule of assessments and online supplementary information 3 for description of the intravitreal procedure.

10.1136/bjophthalmol-2019-315152.supp4 Supplementary data

10.1136/bjophthalmol-2019-315152.supp5 Supplementary data

### Primary outcome measures

There were two primary outcome measures : (i) To report the safety of aflibercept in RP-CMO throughout the study via the documentation of adverse events (AEs) deemed related to the trial drug; (ii) To report the efficacy of aflibercept in RP-CMO via mean CMT on SDOCT at 12 months after baseline.

### Secondary outcome measures

Please refer to online supplementary information 4 for a list of secondary outcome measures.

10.1136/bjophthalmol-2019-315152.supp6 Supplementary data

### Sample size

No previous studies have been published for which the sample size could be powered. A sample size of 30 patients was therefore justified on the basis that 30 subjects will provide an estimate of the mean change in CMT from baseline to 12 months with reasonable precision as advocated by Browne^23^ and Hertzog.^24^

### Masking

This was an open-label study and therefore no masking took place.

### Data management and statistical analysis

Please refer to online supplementary information 5 for information on data management and statistical analysis.

10.1136/bjophthalmol-2019-315152.supp7 Supplementary data

## Results

Baseline characteristics and injection frequency for all participants are summarised in supplementary tables 2–6 and supplementary information 6.

10.1136/bjophthalmol-2019-315152.supp8 Supplementary data

10.1136/bjophthalmol-2019-315152.supp9 Supplementary data

10.1136/bjophthalmol-2019-315152.supp10 Supplementary data

10.1136/bjophthalmol-2019-315152.supp11 Supplementary data

10.1136/bjophthalmol-2019-315152.supp12 Supplementary data

10.1136/bjophthalmol-2019-315152.supp13 Supplementary data

### Outcome measures

#### Efficacy: analysis of all study participants

The primary and secondary efficacy outcomes for all patients (responders and non-responders) within the study are summarised in tables 1 and 2. Mean CMT at 12 months was 413.4 µm (SD 98.2 µm, 95% CI 376.0 to 450.7 µm), corresponding to a reduction in CMT of 47.6 µm (SD 86.6 µm, 95% CI −80.5 to −14.6 µm) or 9.61% (17.56 %) between baseline and 12 months. Mean macular volume at 12 months was 8.0 mm^3^ (SD 0.7, 95% CI 7.7 to 8.2), corresponding to a change in macular volume of −0.3 mm^3^ (SD 0.7, 95% CI −0.6 to −0.1) between baseline and 12 months. Mean CMT at 6 months was similar at 414.8 µm (SD 96.4 µm, 95% CI 378.1 to 451.4 µm), corresponding to a reduction in CMT of 46.2 µm (SD 108.7 µm, 95% CI −87.6 to −4.9 µm) or 8.13% (23.3%) (see supplementary figure 2) between baseline and 6 months. Mean macular volume at 6 months was 7.9 mm^3^ (SD 0.6, 95% CI 7.7 to 8.2), corresponding to a change in macular volume of −0.3 mm^3^ (SD 0.8, 95% CI −0.7 to 0.0) between baseline and 6 months.

10.1136/bjophthalmol-2019-315152.supp14 Supplementary data

**Table 1:** Primary outcome measures

	Aflibercept (n=29)	95% CI
Central macular thickness on SDOCT (µm), mean (SD) at baseline	458.7 (84.6)	
Central macular thickness on SDOCT (µm), mean (SD) at 12 months	413.4 (98.2)	376.0 to 450.7

SDOCT, Spectral domain optical coherence tomography.

**Table 2:** Secondary outcome measures

	Eylea (n=29)	95% CI
Central macular thickness on SDOCT (µm), mean (SD) at 6 months	414.8 (96.4)	378.1 to 451.4
Change in central macular thickness on SDOCT (µm), from		
Baseline to 12 months, mean (SD)	−47.6 (86.6)	−80.5 to −14.6
Baseline to 6 months, mean (SD)	−46.2 (108.7)	−87.6 to −4.9
ETDRS BCVA (letters), mean (SD) at 6 months	66.9 (10.6)	62.8 to 70.9
ETDRS BCVA (letters), mean (SD) at 12 months	68.0 (11.1)	63.8 to 72.3
Change in ETDRS BCVA (letters) from		
Baseline to 12 months, mean (SD)	4.3 (6.9)	1.7 to 6.9
Baseline to 6 months, mean (SD)	3.1 (6.6)	0.6 to 5.6
Macular volume on SDOCT (mm^3^), mean (SD) at 6 months	7.9 (0.6)	7.7 to 8.2
Macular volume on SDOCT (mm^3^), mean (SD) at 12 months	8.0 (0.7)	7.7 to 8.2
Change in macular volume on SDOCT (mm^3^) from		
Baseline to 12 months, mean (SD)	−0.3 (0.7)	−0.6 to −0.1
Baseline to 6 months, mean (SD)	−0.3 (0.8)	−0.7 to 0.0
Retinal sensitivity (dB), mean (SD) at 6 months	4.92 (3.49)	3.56 to 6.27
Missing, n(%)	1 (3)	
Retinal sensitivity (dB), mean (SD) at 12 months	4.93 (3.48)	3.55 to 6.31
Missing, n(%)	2 (6)	
Change in retinal sensitivity (dB) from		
Baseline to 12 months, mean (SD)	−1.09 (2.10)	−1.90 to −0.27
Baseline to 6 months, mean (SD)	−1.23 (2.24)	−2.10 to −0.37
Total number of injections received over the study period (12 months), median (IQR)	7 (6,9)	

BCVA, best-corrected visual acuity; dB, decibels; SDOCT, spectral domain optical coherence tomography.

Mean ETDRS BCVA was 66.9 letters (SD 10.6, 95% CI 62.8 to 70.9) at 6 months and 68.0 letters (SD 11.1, 95% CI 63.8 to 72.3) at 12 months. This equated to a gain of 3.1 letters (SD 6.6, 95% CI 0.6 to 5.6) and 4.3 letters (SD 6.9, 95% CI 1.7 to 6.9) respectively at 6 and 12 months (see supplementary figure 3). No patients lost ≥30 letters.

10.1136/bjophthalmol-2019-315152.supp15 Supplementary data

Mean retinal sensitivity at 6 months was 4.92 dB (SD 3.49, 95% CI 3.56 to 6.27), corresponding to a change in retinal sensitivity of −1.23 dB (SD 2.24, 95% CI −2.1 to −0.37). Data were missing for 1 (3%) patient. Mean retinal sensitivity at 12 months was 4.93 dB (SD 3.48, 95% CI 3.55 to 6.31), corresponding to a change in retinal sensitivity of −1.09 dB (SD 2.10, 95% CI −1.9 to −0.27). Data were missing for 2 (6%) patients.

#### Efficacy: subgroup analysis of responders only

**Figure 1:**
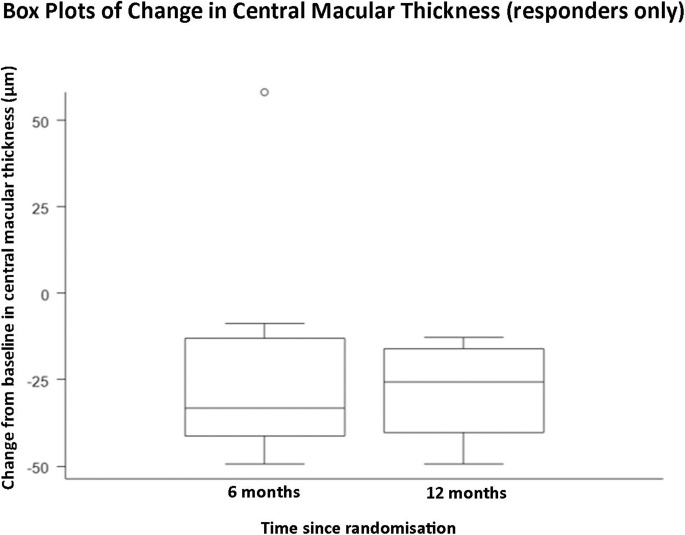
A graph demonstrating mean change in central macular thickness from baseline to 6 months post-baseline, and baseline to 12 months post-baseline in responders only (n=11).

**Figure 2:**
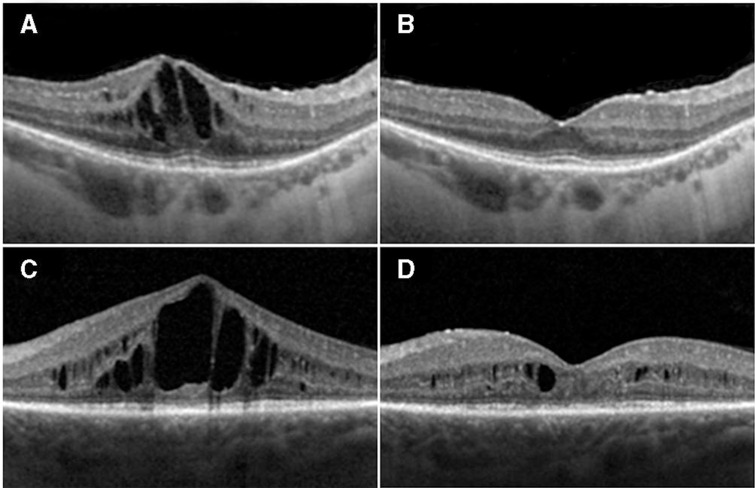
Two representative examples of responders: (A) and (C) show spectral domain optical coherence tomography (SDOCT) baseline images of two study participants (study IDs: 04 and 14); (B) and (D) are SDOCT images taken at 1 month post-first aflibercept injection in the same two participants, respectively

**Figure 3:**
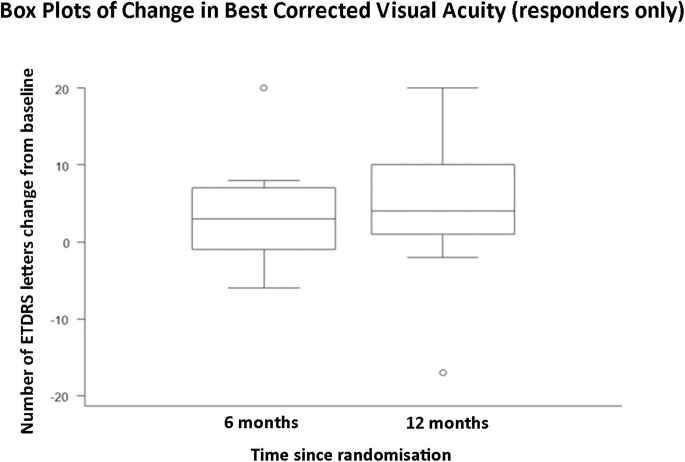
A graph demonstrating mean change in central macular thickness from baseline to 6 months post-baseline, and baseline to 12 months post-baseline in responders only (n=11).

The primary and secondary efficacy outcomes using descriptive statistics for subgroup analysis of responders within the study are provided in table 3, figures 1–3 and supplementary information 7.

10.1136/bjophthalmol-2019-315152.supp16 Supplementary data

**Table 3:** Descriptive statistics for responders

	Eylea(n=11)
Central macular thickness on SDOCT (µm), mean (SD) at 12 months	350.3 (93.3)
Central macular thickness on SDOCT (µm), mean (SD) at 6 months	360.7 (85.2)
Change in central macular thickness on SDOCT (µm) from	
Baseline to 12 months, mean (SD)	–139.5 (65.8)
Baseline to 6 months, mean (SD)	–129.1 (125.1)
ETDRS BCVA (letters), mean (SD) at 6 months	67.5 (10.1)
ETDRS BCVA (letters), mean (SD) at 12 months	68.4 (11.8)
Change in ETDRS BCVA (letters) from	
Baseline to 12 months, mean (SD)	4.7 (9.5)
Baseline to 6 months, mean (SD)	3.8 (6.8)
Macular volume on SDOCT (mm^3^), mean (SD) at 6 months	8.5 (0.6)
Macular volume on SDOCT (mm^3^), mean (SD) at 12 months	8.5 (0.8)
Change in macular volume on SDOCT (mm^3^) from	
Baseline to 12 months, mean (SD)	–0.6 (0.6)
Baseline to 6 months, mean (SD)	–0.6 (0.6)
Retinal sensitivity (dB), mean (SD) at 6 months	4.93 (4.06)
Retinal sensitivity (dB), mean (SD) at 12 months	4.48 (3.83)
Change in retinal sensitivity (dB) from	
Baseline to 12 months, mean (SD)	–0.97 (1.92)
Baseline to 6 months, mean (SD)	–0.92 (2.03)
Total number of injections received over the study period (12 months), median (IQR)	7 (6,10)

BCVA, best corrected visual acuity; dB, decibels; SDOCT, spectral domain optical coherence tomography.

#### Safety: analysis of all study participants

Ocular and non-ocular AEs and serious AEs are summarised in online supplementary tables 7-9 and online supplementary information 8.

10.1136/bjophthalmol-2019-315152.supp17 Supplementary data

10.1136/bjophthalmol-2019-315152.supp18 Supplementary data

10.1136/bjophthalmol-2019-315152.supp19 Supplementary data

10.1136/bjophthalmol-2019-315152.supp20 Supplementary data

## Discussion

This is the first prospective study to obtain safety and efficacy data over a 12-month period using serial intravitreal injections with aflibercept for the treatment of RP-CMO. There were no significant safety concerns and serial injections were well-tolerated. Eleven out of 29 (37.9%) patients were classified as responders at both 6 and 12 months having demonstrated a reduction of at least 11% CMT on SDOCT compared with baseline. These patients experienced a mean (SD) percentage change in CMT relative to baseline of −22.9% (29.7 %) and −28.1% (12.9 %) at 6 and 12 months respectively. Responders gained 3.8 (SD 6.8) and 4.7 (SD 9.5) ETDRS letters respectively at 6 and 12 months. Responders demonstrated a greater change of macular volume over the study (−0.6 mm^3^ at 6 and 12 months) compared with non-responders (−0.3 mm^3^ at 6 and 12 months). When the cohort was analysed as a whole, the mean (SD) percentage change in CMT relative to baseline was −8.1% (23.3%) and −9.6% (17.6%) at 6 and 12 months respectively. An intriguing observation, unlike other disorders where anti-VEGF agents have been employed, is that all responders (n=11) achieved a notable reduction in CMO after their first injection (‘early-responder’, Figure 2). There were no ‘late-responders’. This is clinically very valuable as for the majority of patients it may be possible to decide at a very early stage whether injections should be pursued.

Responders in this study were identified across all categories of inheritance pattern (AD, AR and XL). There was no association between response to anti-VEGF treatment and mode of inheritance. While just over half of the patients in this study had a confirmed molecular diagnosis, no specific genotype was associated with response to treatment (for example, one USH2A patient responded, two others did not; one PRPF31 patient responded, two others did not). This study included only one patient with XL inheritance who was deemed a responder and we therefore cannot draw any comparison with other patients with XL-RP. More advanced disease, defined as disruption of the ellipsoid zone within 1 mm of the fovea (seen in 27.3% of responders and 33.3% of non-responders) did not affect likelihood of response to anti-VEGF.

The release of toxic products (including VEGF) from degenerating retina/RPE in patients with RP contributes to blood-retinal barrier weakening and RP-CMO formation.^7^ Anti-VEGF is thought to act by reversing proliferation and cell migration stimulated by VEGF and the delocalisation of tight junction proteins induced by VEGF165.^25^ Intriguingly, Salom *et al* observed lower aqueous levels of VEGF in eyes of patients with RP versus controls.^26^ It would be interesting to measure levels of VEGF in the vitreous and review whether there are significant differences between patients with RP versus controls, as well as patients with RP versus those with RP-CMO. This being an invasive procedure, however, would likely prove challenging to gain ethical approval and is why we did not consider undertaking in this study.

Oxidative stress may also play a role in the development of CMO. In the case of diabetic retinopathy, raised circulating blood sugar is thought to cause dysregulation of several biochemical and molecular signalling pathways leading to the production of superoxide-free radicals and resultant oxidative stress in retinal tissues.^27^ Mitochondrial dysfunction, inflammation, and hypoxia-driven VEGF release leads to vascular and neuronal apoptosis and neovascularisation and elevated vasopermeability, respectively.^27^

Animal models of RP have demonstrated increased production of superoxide-free radicals due to elevated oxygen levels in the outer retina. This occurs because, despite rod photoreceptor death and therefore reduced oxygen requirements, the choroid continues to supply the retina with the same blood flow and oxygen delivery.^28^ A study by Campochiaro *et al* demonstrated ocular oxidative stress in patients with RP in the absence of manifestations of systemic oxidative stress and/or damage.^29^ It is therefore possible that oxidative damage-induced cone cell death in animal models of RP may translate to human RP. Antioxidants may therefore promote cone survival and function of patients with RP.^29^ They may also influence RP-CMO.

Strengths of our study include excellent patient attendance throughout its duration, with a 96.7% participant retention rate at 12 months. The study drug was well-tolerated and no cases of endophthalmitis occurred. The study design including an initial loading phase followed by a treat and-extend regime, which allowed for the observation of both early and (potentially) late responders. We also established likely disease-causing sequence variants in 16 of 30 (53.3%) study participants.

Patients were reluctant to receive intravitreal injections without first trialling topical and/or oral treatment. A limitation to our study was therefore being unable to include treatment-naive patients with shorter duration of CMO. All patients in the study had used topical CAI medication previously; 15 of whom were using topical CAI treatment up until 1 month prior to their screening appointment. Five of these patients were deemed responders. Five patients in the study were using oral CAI treatment up until 3 months prior to their screening appointment; one patient withdrew from the study, two patients were deemed responders, and two patients did not respond. No obvious trend was demonstrated to suggest whether recent use of topical or oral CAIs influences response to anti-VEGF therapy.

Long-standing CMO duration was observed in many patients within our cohort, with the median duration being 252 weeks (IQR, 156–296 weeks). Interestingly, duration of CMO did not appear to affect anatomical response to anti-VEGF; median CMO duration in responders was 264 weeks (IQR 228, 416), compared with the group overall (252 weeks (IQR 156, 296). In fact, the patient with the longest-standing CMO duration of the cohort (20 years) had complete resolution of CMO after a single ivA.

Our study included patients with fairly advanced underlying disease as demonstrated by photoreceptor loss and outer retinal thinning—features that have been shown to hinder VA improvement despite reduction of CMT.^30^ Indeed, three of 11 (27.3%) responders graded as having disruption of the ellipsoid zone within 1 mm of the fovea on their baseline OCT scan demonstrated no improvement of vision. Greater improvement of VA may be demonstrated in patients with a relatively more intact photoreceptor layer at baseline.

It would be valuable to repeat this study in a larger cohort of patients with molecularly confirmed genetic diagnosis, ideally naive to other treatment modalities, shorter history of CMO duration and relatively intact photoreceptor layer at baseline. Additional suggestions include: baseline fundus fluorescein angiogram to see whether active leakage is present and whether this predicts likelihood of response to aflibercept, baseline vitreous samples to assess VEGF levels, inclusion of a control group (possibly using placebo), randomisation of patients, to blind patients and/or clinicians and to include OCT-angiography as an additional imaging modality.

This phase II exploratory study demonstrates that ivA can be effective at reducing CMT in patients with RP-CMO, however, the factors predicting who is likely to respond remain to be clarified. There may be a role in considering intravitreal aflibercept as part of the future armamentarium when selecting treatments for patients with RP-CMO, particular when chronic and unresponsive to alternative treatments. A larger study is required to obtain additional safety data and further investigate the role of VEGF blockade in RP-CMO.
