# Supplementary figures and images for: Prospective exploratory study to assess the safety and efficacy of aflibercept in cystoid macular oedema associated with retinitis pigmentosa
Source: Br J Ophthalmol. 2020 Sep 1;104(9):1203–8. doi: 10.1136/bjophthalmol-2019-315152 (PMC7577098; doi:10.1136/bjophthalmol-2019-315152)

**Supplementary figure 1: Consort flow diagram**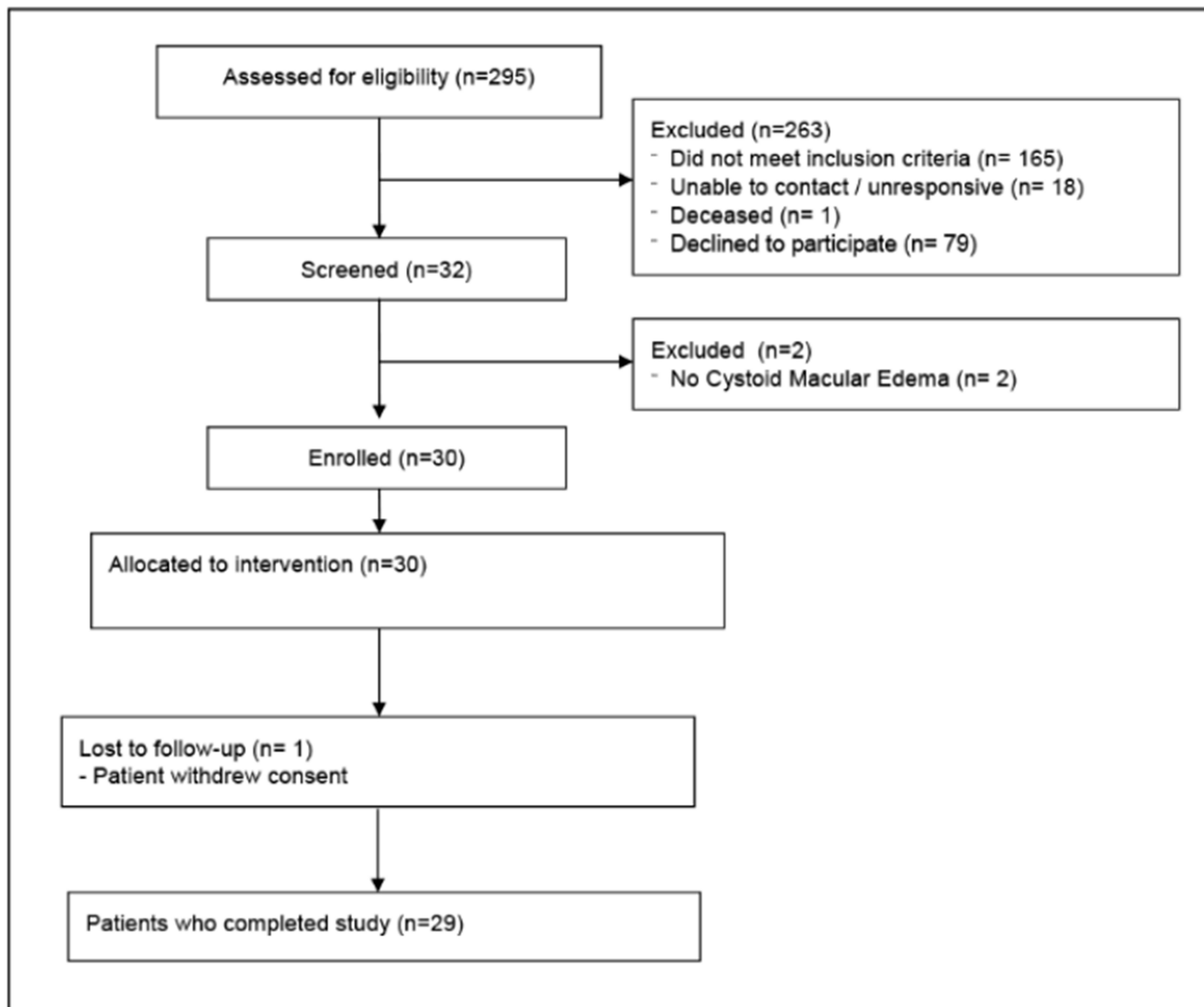

Supplement: Supplementary data [file bjophthalmol-2019-315152s001.pdf]
