# Supplementary material for: Prospective exploratory study to assess the safety and efficacy of aflibercept in cystoid macular oedema associated with retinitis pigmentosa
Source: Br J Ophthalmol. 2020 Sep 1;104(9):1203–8. doi: 10.1136/bjophthalmol-2019-315152 (PMC7577098; doi:10.1136/bjophthalmol-2019-315152)
Supplement: Supplementary data [file bjophthalmol-2019-315152s002.pdf]

Patient eligibility

The following criteria were used to guide patient enrollment:

(A) *Inclusion criteria*: (1) patients of either gender aged ≥16 years; (2) CMO in association with RP; (3) Unilateral or Bilateral CMO (the worse eye only treated – defined as the eye with a greater central macular thickness (CMT) on OCT); (4) No previous oral treatment for CMO for last 3 months; (5) No previous peribulbar or intravitreal treatment for CMO in the study eye for last 3 months; (6) No previous topical treatment for CMO in the study eye for last 1 month; (7) Central visual impairment that in the view of the Principal Investigator (PI) was due to CMO; (8) BCVA better than 20/400.

(B) *Exclusion criteria* (ocular criteria were applied to the *study eye only*): (1) Insufficient patient cooperation or media clarity to allow adequate fundus imaging; (2) Evidence of visually significant vitreo-retinal traction or epiretinal membrane (ERM) on OCT that in the PI's opinion was likely to significantly limit the efficacy of intravitreal therapy; (3) History of cataract surgery within prior 3 months or cataract surgery anticipated within 6 months of starting the study; (4) Any anti-VEGF treatment to study eye within 3 months; (5) History of YAG capsulotomy performed within 3 months; (6) Uncontrolled IOP > = 24 mmHg for ocular hypertension (on topical IOP lowering medications); (7) Advanced glaucoma (in the opinion of a glaucoma specialist); (8) Patients with active or suspected ocular or periocular infections; (9) Patients with active severe intraocular inflammation; (10) Patients with a new, untreated retinal tear or detachment; (11) Patients with a stage 3 or 4 macular hole; (12) Thromboembolic event (MI/CVA/Unstable Angina) within 6 months; (13) Pregnancy or family planned within 15 months; (14) Breast feeding; (15) Known allergy or hypersensitivity to anti-VEGF products.
