# Supplementary material for: Prospective exploratory study to assess the safety and efficacy of aflibercept in cystoid macular oedema associated with retinitis pigmentosa
Source: Br J Ophthalmol. 2020 Sep 1;104(9):1203–8. doi: 10.1136/bjophthalmol-2019-315152 (PMC7577098; doi:10.1136/bjophthalmol-2019-315152)
Supplement: Supplementary data [file bjophthalmol-2019-315152s003.pdf]

Baseline tests of visual function

Subjective refraction and best-corrected visual acuity (BCVA)

All patients were subjectively refracted at baseline to obtain their spectacle correction. BCVA was tested monocularly at 4 metres (m) using 2 ETDRS charts (one chart for each eye) that were retro-illuminated using a light box containing 2 Cool Daylight 20 watt fluorescent tubes. If a patient was unable to read 20 letters or more at this distance, the test would be repeated at 1m. In this case, only the first 6 rows would be attempted. If the patient was wearing a trial frame, +0.75 dioptres sphere (DS) was added to the prescription to correct for the closer test distance. The VA score was the number of letters read correctly at 4m, plus the number of letters read correctly at 1m. If the patient did not require testing at 1m, i.e. they read 20 or more letters at 4m, then the score was the number of letters read correctly at 4m, plus 30.

Spectral Domain Optical Coherence Tomography (SDOCT)

The Heidelberg Spectralis SDOCT (Heidelberg Engineering, Heidelberg, Germany) was used to obtain macular volume scans in order to measure CMT. The macular volume scan protocol had the following settings: 20°x20°, 49 Sections, High Speed, 29 frames automatic real time (ART).

Ishihara colour vision testing

The Ishihara version used contained 17 plates held at 75 cm from the patient. A light box was used to achieve standardised lighting and patients were adequately corrected for reading vision using plus lenses if required performing the test monocularly.

Contrast sensitivity

Contrast sensitivity was performed monocularly using the Pelli-Robson chart (Clement Clarke Inc., Columbus, OH). The patient was seated at a distance of 1m. As a standard, +0.75DS was added to each patient's refraction when performing the test. The luminance of the chart was between 80 - 120 candela per square metre (cd/m^2). The patient was asked to name each letter on the chart, starting with the high-contrast letters on the upper left-hand corner and reading horizontally across the entire line. The test was completed when the patient failed to correctly identify two or more letters in a triplet.

Retinal sensitivity

Mesopic microperimetry using the MP-1 microperimeter (Nidek Instruments, Inc, Padua, Italy) was carried out *twice* on each eye at baseline. Spherical error was accounted for in all patients who were then dark-adapted for 10 minutes before performing the test. The microperimetry protocol included: Cross 2 degrees, Goldmann Ill Stimulus 200ms, 4-2 strategy and 30 seconds of tracked fixation. The results were generated using a local defect map including -9:1 setting in order to provide mean sensitivity and mean defect together with bivariate contour ellipse area (BCEA) value (numeric and fixation).

Fundus autofluorescence (FAF)

FAF images were acquired using the Heidelberg Spectralis. For each eye, a near-infrared reflectance image and short-wavelength autofluorescence image of standard field 2 (centred on the fovea) were acquired. Both 30 and 55 degree field of view were acquired using high resolution at 50 frames (ART).
