# Supplementary material for: Prospective exploratory study to assess the safety and efficacy of aflibercept in cystoid macular oedema associated with retinitis pigmentosa
Source: Br J Ophthalmol. 2020 Sep 1;104(9):1203–8. doi: 10.1136/bjophthalmol-2019-315152 (PMC7577098; doi:10.1136/bjophthalmol-2019-315152)
Supplement: Supplementary data [file bjophthalmol-2019-315152s004.pdf]

Supplementary table 1: Schedule of assessments

	Screening and Baseline	Treatment phase and follow-up*											
Visit number	1	2	3	4	5	6	7	8	9	10	11	12	13
Weeks	0 weeks	4 weeks post baseline	8 weeks post baseline	4 to 12 weeks post visit 3	4 to 12 weeks post visit 4	4 to 12 weeks post visit 5	4 to 12 weeks post visit 6	4 to 12 weeks post visit 7	4 to 12 weeks post visit 8	4 to 12 weeks post visit 9	4 to 12 weeks post visit 10	4 to 12 weeks post visit 11	4 to 12 weeks post visit 12
Patient demographics confirmed	X	X	x	X	x	x	x	x	x	x	x	x	X
General medical and ocular history	X												
Medication review	X	x	x	x	x	x	x	x	x	x	x	x	X
Pregnancy status confirmed	X												
Eligibility check	X												
Informed Consent	X												
Vital signs: Blood pressure, heart rate and temperature	X	X	x	X	x	x	x	x	x	x	x	x	X
Best corrected ETDRS visual acuity	X	X	X	X	x	x	x	x	x	x	x	x	x
Refracted best corrected ETDRS visual acuity	X					X (at 6 months)							X (at 12 months)
Colour vision	X	X	X	X	x	x	x	x	x	x	x	x	x
Contrast sensitivity	X	X	X	X	x	x	x	x	x	x	x	x	x
Microperimetry	X					X (at 6 months)							X (at 12 months)
Dilation of the patient	X	X	X	X	x	x	x	x	x	x	x	x	x
Slit lamp examination	X	X	X	X	x	x	x	x	x	x	x	x	x
IOP check (pre-injection)	X	X	X	X	x	x	x	x	x	x	x	x	x
SDOCT in both eyes	X	X	X	X	x	x	x	x	x	x	x	x	x
Fundus Autofluorescence	X	X	X	X	x	x	x	x	x	x	x	x	x
Administration of Eylea	Possibly	X	X	X	x	x	x	x	x	x	x	x	x
IOP check (post-injection)	X	X	X	X	x	x	x	x	x	x	x	x	x

***Number of total visits will vary between patients as follow-up appointments will be any time between 4 to 12 weeks.**
