# Supplementary material for: Prospective exploratory study to assess the safety and efficacy of aflibercept in cystoid macular oedema associated with retinitis pigmentosa
Source: Br J Ophthalmol. 2020 Sep 1;104(9):1203–8. doi: 10.1136/bjophthalmol-2019-315152 (PMC7577098; doi:10.1136/bjophthalmol-2019-315152)
Supplement: Supplementary data [file bjophthalmol-2019-315152s005.pdf]

***Intravitreal Procedure***

Aflibercept (Eylea; Regeneron, Tarrytown, New York, USA and Bayer Healthcare, Leverkusen, Germany) was supplied by Bayer LTD in vials containing 100 microlitres of 40mg/ml solution for injection, equivalent to 4mg aflibercept and stored by Moorfields Pharmaceuticals (London, UK). Each vial enabled a usable amount to deliver a single dose of 50 microlitres containing 2 mg aflibercept. In a designated intravitreal treatment room, under sterile conditions, using topical anesthesia and povidone-iodine 5% into the conjunctival sac and onto the lid margins, and following application of a drape and insertion of a lid speculum, injections were undertaken with a 30-gauge needle through the infratemporal quadrant, with a drop of preservative-free (PF) chloramphenicol placed in the fornix at the end of the procedure. Patency of the central retinal artery was determined by visual acuity (VA) of hand movements or better. After the injection, topical chloramphenicol was self-instilled 4 times per day for 5 days by the patients.
