# Supplementary material for: Prospective exploratory study to assess the safety and efficacy of aflibercept in cystoid macular oedema associated with retinitis pigmentosa
Source: Br J Ophthalmol. 2020 Sep 1;104(9):1203–8. doi: 10.1136/bjophthalmol-2019-315152 (PMC7577098; doi:10.1136/bjophthalmol-2019-315152)
Supplement: Supplementary data [file bjophthalmol-2019-315152s006.pdf]

Secondary outcome measures

The secondary outcome measures relating to efficacy were: (i) The mean CMT on SDOCT at 6 months after baseline; (ii) The mean change in CMT on SDOCT from baseline to 6 months after baseline and baseline to 12 months after baseline; (iii) The mean Best Corrected Visual Acuity (BCVA) using the ETDRS visual acuity chart at a starting distance of 4m at 6 and 12 months after baseline; (iv) The mean change in ETDRS BCVA from baseline to 6 months and baseline to 12 months; (v) The mean macular volume on SDOCT at 6 and 12 months; (vi) The mean change in macular volume on SDOCT from baseline to 6 months and baseline to 12 months; (vii) Report all AEs and serious adverse events (SAEs) throughout the study; (viii) The mean retinal sensitivity using microperimetry at 6 and 12 months; (ix) The mean change in retinal sensitivity using microperimetry from baseline to 6 months and baseline to 12 months; (x) The mean number of intravitreal injections administered throughout the study.
