# Supplementary material for: Prospective exploratory study to assess the safety and efficacy of aflibercept in cystoid macular oedema associated with retinitis pigmentosa
Source: Br J Ophthalmol. 2020 Sep 1;104(9):1203–8. doi: 10.1136/bjophthalmol-2019-315152 (PMC7577098; doi:10.1136/bjophthalmol-2019-315152)
Supplement: Supplementary data [file bjophthalmol-2019-315152s007.pdf]

Supplementary Information 5

Data management

The completed paper case report forms (CRFs) were checked for completion by the research nurse / research manager and data officer before data entry. All trial data were double entered by two independent data officers using the database created by the R&D IT team. The first and second data entries were compared for completion and consistency. Discrepancies were checked against the original CRF for entry errors, which were subsequently corrected. Sense checks, logic checks and range checks were also performed. Data queries were corrected and data were cleaned. The database was then locked and data transferred for data to be analysed by trial statisticians using STATA statistical software. The data management process followed Moorfields Eye Hospital standard operating procedures (SOPs) for data management.

Statistical analysis

The primary analysis was an available case analysis but baseline characteristics of those who were lost to follow up were compared with those who were not. If the findings from this study were favourable, these data would be used to plan a definitive future randomised controlled trial.
Descriptive statistics have been used to report the findings of this study due to its modest sample size and single arm design. 'Responders' would be considered as participants demonstrating a reduction of CMT by 11% or more between baseline and 12 months, thus allowing comparison with previous studies that have used the same definition [1-6] . All statistical analyses were conducted using Stata Statistical Software version 15.0.

References:

1. Liew G, Moore AT, Webster AR, et al. Efficacy and prognostic factors of response to carbonic anhydrase inhibitors in management of cystoid macular edema in retinitis pigmentosa. Invest Ophthalmol Vis Sci 2015;56:1531-1536.

2. Kitahata S, Hirami Y, Takagi S, et al. Efficacy of additional topical betamethasone in persistent cystoid macular oedema after carbonic anhydrase inhibitor treatments in retinitis pigmentosa. BMJ Open Ophthalmol 2018;3:e000107.

3. Lemos Reis RF, Moreira-Goncalves N, Estrela Silva SE, et al. Comparison of topical dorzolamide and ketorolac treatment for cystoid macular edema in retinitis pigmentosa and Usher's syndrome. Ophthalmologica 2015;233:43-50.

4. Tripathy K. Cystoid Macular Edema in Retinitis Pigmentosa with Intermediate Uveitis Responded Well to Oral and Posterior Subtenon Steroid. Semin Ophthalmol 2018;33:492-493.

5. Grover S, Apushkin MA, Fishman GA. Topical dorzolamide for the treatment of cystoid macular edema in patients with retinitis pigmentosa. Am J Ophthalmol 2006;141:850-858.

6. Genead MA, Fishman GA. Efficacy of sustained topical dorzolamide therapy for cystic macular lesions in patients with retinitis pigmentosa and usher syndrome. Arch Ophthalmol 2010;128:1146-1150.
