# Supplementary material for: Prospective exploratory study to assess the safety and efficacy of aflibercept in cystoid macular oedema associated with retinitis pigmentosa
Source: Br J Ophthalmol. 2020 Sep 1;104(9):1203–8. doi: 10.1136/bjophthalmol-2019-315152 (PMC7577098; doi:10.1136/bjophthalmol-2019-315152)
Supplement: Supplementary data [file bjophthalmol-2019-315152s008.pdf]

Supplementary table 2: Genetic data of all patients in the study

Study ID	Symbol	Protein	Inheritance pattern
1	PFPR31	pre-MRNA processing factor 31	AD
2	PFPR8	pre-MRNA processing factor 8	AD
3	USH2A<br>Location:<br>c.1841-2A>G<br>homozygous	usherin 2A	AR
4	Unsolved	Unsolved	Unsolved
5	RP11	retinitis pigmentosa-11	AD
6	RP1	retinitis pigmentosa-1	AD
7	TULP1	tubby like protein 1	AR
8	Unsolved	Unsolved	Unsolved
9	NRL	neural retina leucine zipper	AD
10	IFT140	intraflagellar transport-140	AR
11	Unsolved	Unsolved	Unsolved
12	RHO/RP4	rhodopsin	AD
13	Unsolved	Unsolved	Unsolved
14	Unsolved	Unsolved	Unsolved
15	RHO	rhodopsin	AD
16	Unsolved	Unsolved	Unsolved
17	Unsolved	Unsolved	Unsolved
18	SNRNP200	small nuclear ribonucleoprotein U5 subunit 200	AR
19	Unsolved	Unsolved	Unsolved
20	USH2A<br>Location:<br>c.2299del,<br>p.Glu767Serf<br>s*21 hom	usherin 2A	AR
21	Unsolved	Unsolved	Unsolved

22	Unsolved	Unsolved	Unsolved
23	Unsolved	Unsolved	Unsolved
24	PRPF31	pre-MRNA processing factor 31	AD
25	Unsolved	Unsolved	Unsolved
26	Unsolved	Unsolved	Unsolved
27	Unsolved	Unsolved	Unsolved
28	USH2A Location: c.11700C>A, p.Tyr3900Ter; c.4618G>A, p.Asp1540Asn	usherin 2A	AR
29	n/a	n/a	n/a
30	RPGR	retinitis pigmentosa GTPase regulator	XL
31	n/a	n/a	n/a
32	RDH12	retinol dehydrogenase-12	AD
