# Supplementary material for: Prospective exploratory study to assess the safety and efficacy of aflibercept in cystoid macular oedema associated with retinitis pigmentosa
Source: Br J Ophthalmol. 2020 Sep 1;104(9):1203–8. doi: 10.1136/bjophthalmol-2019-315152 (PMC7577098; doi:10.1136/bjophthalmol-2019-315152)
Supplement: Supplementary data [file bjophthalmol-2019-315152s009.pdf]

Supplementary table 3: Non-Ocular Baseline Characteristics

	Aflibercept
Number of Patients (Eyes)	30 (30)
Male / Female, n (%)	17 (57)/ 13(43)
Age (years), Mean (SD)	43.3 (11.5)
Ethnicity, n (%):	
White	26 (87)
Asian	1 (3)
Black	1 (3)
Mixed	1 (3)
Other	1 (3)
