# Supplementary material for: Prospective exploratory study to assess the safety and efficacy of aflibercept in cystoid macular oedema associated with retinitis pigmentosa
Source: Br J Ophthalmol. 2020 Sep 1;104(9):1203–8. doi: 10.1136/bjophthalmol-2019-315152 (PMC7577098; doi:10.1136/bjophthalmol-2019-315152)
Supplement: Supplementary data [file bjophthalmol-2019-315152s010.pdf]

Supplementary table 4: Ocular Baseline Characteristics

| | Aflibercept (n=30) |
|---|---|
| Study Eye, Left/Right, n(%) | 16 (53)/ 14 (47) |
| Duration of CME (weeks), Median (IQR) | 252 (156 to 296) |
| Lens status, n (%): | |
| Aphakic | 0 (0) |
| Pseudophakic | 6 (20) |
| Phakic | 24 (80) |
| ETDRS BCVA (letters), Mean (SD) | 64 (11.5) |
| Ishihara colour vision (out of 17 plates), Median (IQR) | 15 (6-16) |
| Contrast sensitivity ($cd/m^2$) , Mean (SD) | 1.58 (0.35) |
| IOP (mmHg), Mean (SD) | 12.5 (2.9) |
| Central macular thickness on SDOCT (µm), Mean (SD) | 458.7 (84.6) |
| Macular Volume on SDOCT ($mm^3$), Median (IQR) | 8.0 (7.5 to 8.8) |
| Mean Retinal sensitivity on microperimetry (dB), Mean (SD) | 6.3 (3.6) |

CME = cystoid macular edema; µm = microns; ETDRS = early treatment diabetic retinopathy study; BCVA = best corrected visual acuity; SD = standard deviation; IQR = Interquartile range; $cd/m^2$ = candela per square meter; IOP = intraocular pressure; mmHg = millimetre of mercury; SDOCT = Spectral domain optical coherence tomography; $mm^3$ = millimetres cubed; dB = decibels
