# Supplementary material for: Prospective exploratory study to assess the safety and efficacy of aflibercept in cystoid macular oedema associated with retinitis pigmentosa
Source: Br J Ophthalmol. 2020 Sep 1;104(9):1203–8. doi: 10.1136/bjophthalmol-2019-315152 (PMC7577098; doi:10.1136/bjophthalmol-2019-315152)
Supplement: Supplementary data [file bjophthalmol-2019-315152s011.pdf]

Supplementary table 5: Non-Ocular Baseline Characteristics (Responders only)

	Aflibercept
Number of Patients (Eyes)	11 (11)
Male / Female, n (%)	8 (73)/3(27)
Age (years), Mean (SD)	42.7 (15.6)
Ethnicity, n (%):	
White	11 (100)
Asian	0 (0)
Black	0 (0)
Mixed	0 (0)
Other	0 (0)
