# Supplementary material for: Prospective exploratory study to assess the safety and efficacy of aflibercept in cystoid macular oedema associated with retinitis pigmentosa
Source: Br J Ophthalmol. 2020 Sep 1;104(9):1203–8. doi: 10.1136/bjophthalmol-2019-315152 (PMC7577098; doi:10.1136/bjophthalmol-2019-315152)
Supplement: Supplementary data [file bjophthalmol-2019-315152s012.pdf]

Supplementary table 6: Ocular Baseline Characteristics (Responders only)

	Aflibercept (n=11)
Study Eye, Left/Right, n(%)	6 (55)/5 (45)
Duration of CME (weeks), Median (IQR)	264 (228, 416)
Lens status, n (%):	
Aphakic	0 (0)
Pseudophakic	1 (9)
Phakic	10 (91)
ETDRS BCVA (letters), Mean (SD)	63.6 (11.3)
Ishihara colour vision (out of 17 plates), Median (IQR)	10 (3 to 14)
Contrast sensitivity (cd/m^2), Mean (SD)	1.42 (0.38)
IOP (mmHg), Mean (SD)	12.4 (3.4)
Central macular thickness on SDOCT (µm), Mean (SD)	489.8 (105.9)
Macular Volume on SDOCT (mm^3),Median (IQR)	8.9 (8.3 to 9.9)
Mean Retinal sensitivity on microperimetry (dB), Mean (SD)	5.8 (3.7)

CME = cystoid macular edema; µm = microns; ETDRS = early treatment diabetic retinopathy study; BCVA = best corrected visual acuity; SD = standard deviation; IQR = Interquartile range; cd/m^2 = candela per square meter; IOP = intraocular pressure; mmHg = millimetre of mercury; SDOCT = Spectral domain optical coherence tomography; mm^3 = millimetres cubed; dB = decibels
