# Supplementary material for: Prospective exploratory study to assess the safety and efficacy of aflibercept in cystoid macular oedema associated with retinitis pigmentosa
Source: Br J Ophthalmol. 2020 Sep 1;104(9):1203–8. doi: 10.1136/bjophthalmol-2019-315152 (PMC7577098; doi:10.1136/bjophthalmol-2019-315152)
Supplement: Supplementary data [file bjophthalmol-2019-315152s013.pdf]

Baseline characteristics and injection frequency

Thirty eyes of 30 patients were enrolled, with the first patient recruited in March 2016 and the final patient had their 52-week visit in August 2017. Two patients were screened who did not satisfy the criteria for enrolment (study ID 29 and 31); the reason being that they no longer had CME. The mean age of the patients was 43.3 years (SD 11.5 years, range 20 to 61 years), consisting of 17 male (56.7%) and 13 female (43.3%) patients. The study eye was the left eye in 16 (53.3%) patients and the right eye in 14 (47.7%). The median duration of CME in the study eye was 252 weeks and the interquartile range (IQR) was 156 to 296 weeks.

All patients enrolled in the study received the active drug, aflibercept. The median number of injections given across all patients in the study was 7 (IQR 6 to 9); with the minimum number of injections given being 4, and the maximum number of injections given being 11.

Likely disease-causing sequence variants were identified in 16 of 30 (53.3%) study participants (see supplementary table 2), which included: (i) AD inheritance: neural retina leucine zipper (NRL) gene (1 patient), rhodopsin (RHO) gene (2 patients), pre-mRNA processing factor 31 (PRPF31) gene (3 patients), pre-mRNA processing factor-8 (PRPF8) gene (1 patient), small nuclear ribonucleoprotein U5 subunit 200 (SNRNP200) gene (1 patient); (ii) AR inheritance: usherin 2A (USH2A) gene (3 patients), tubby like protein 1 (TULP1) gene (1 patient), retinitis pigmentosa-1 (RP1) gene (1 patient), retinol dehydrogenase-12 (RDH12) gene (1 patient), intraflagellar transport-140 (IFT140) gene (1 patient); and (iii) X-linked inheritance: retinitis pigmentosa GTPase regulator (RPGR) gene (1 patient).

The other 14 patients have undergone genetic screening (including whole genome sequencing) and remain unsolved to date.

Mean baseline ETDRS BCVA was 64 letters (SD 11.5 letters) with a mean CMT of 458.7 microns (SD 84.6 microns) in the study eye for the cohort overall. Twenty-four (80%) patients were phakic, compared with 6 (20%) patients who were pseudophakic in their study eye.

Nine of 29 (31.0%) patients were graded as having either questionable or definite presence of ERM within 3mm of the fovea. No patients were found to have vitreo-macular traction (VMT) on their baseline OCT scan. One of 29 (3.4%) patients was found to have vitreo-macular adhesion on their baseline OCT scan. Nine of 29 (31.0%) patients were graded as having either questionable or definite disruption of the ellipsoid zone within 1mm of the fovea on their baseline OCT scan.

One participant did not complete 12 months of follow-up due to illness and withdrew from the study. Since a single patient only withdrew, analysis was conducted using available case data. The baseline characteristics for this participant who withdrew from the study were not different to patients who continued in the study. Twenty-nine out of 30 (96.7%) patients therefore completed 12 months of follow-up for the study.

A post-hoc exploratory analysis of responders-only was also undertaken. Baseline characteristics for responders are summarized in supplementary tables 5 and 6. Sub-group analysis of responders demonstrated similar baseline characteristics to the group taken as a whole, with mean baseline ETDRS BCVA of 63.6 letters (SD 11.3 letters), mean CMT of 489.8 microns (SD 105.9 microns), and median duration of CME was 264 weeks (IQR 228 to 416) . The median number of injections for this group was 7 (IQR 6 to 10); where the minimum number of injections given was 5, and the maximum number of injections was 11.
