# Supplementary material for: Prospective exploratory study to assess the safety and efficacy of aflibercept in cystoid macular oedema associated with retinitis pigmentosa
Source: Br J Ophthalmol. 2020 Sep 1;104(9):1203–8. doi: 10.1136/bjophthalmol-2019-315152 (PMC7577098; doi:10.1136/bjophthalmol-2019-315152)
Supplement: Supplementary data [file bjophthalmol-2019-315152s015.pdf]

**Supplementary figure 3**

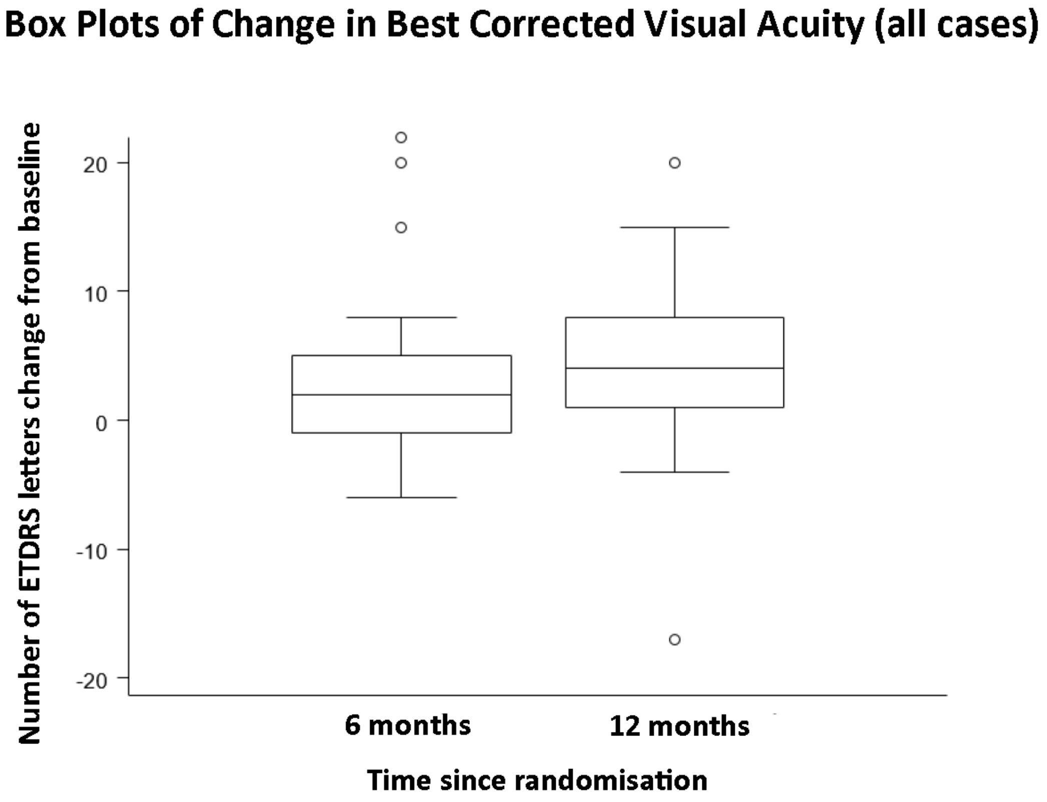


Box plots of change in best corrected visual acuity demonstrating mean change from baseline to 6 and 12 months follow-up visits in the group overall (n=29).

ETDRS: early treatment diabetic retinopathy study
