# Supplementary material for: Prospective exploratory study to assess the safety and efficacy of aflibercept in cystoid macular oedema associated with retinitis pigmentosa
Source: Br J Ophthalmol. 2020 Sep 1;104(9):1203–8. doi: 10.1136/bjophthalmol-2019-315152 (PMC7577098; doi:10.1136/bjophthalmol-2019-315152)
Supplement: Supplementary data [file bjophthalmol-2019-315152s016.pdf]

Efficacy: sub-group analysis of responders only

The primary and secondary efficacy outcomes using descriptive statistics for sub-group analysis of responders within the study are provided in table 3 and supplementary information 7.

Eleven out of 29 (37.9%) patients were classified as responders having demonstrated a reduction in CMT of 11% or more at 12 months compared to baseline. These same patients were also classed as responders at 6 months when applying the same criteria. Genetic mutations were identified and confirmed in 5 of 11 (45.5%) responders, which included: RPGR (1 patient), PRPF31 (1 patient), USH2A (c.11700C>A, p.Tyr3900Ter; c.4618G>A, p.Asp1540Asn) (1 patient), RHO (1 patient) and RDH12 (1 patient). The other 6 patients are genetically unsolved to date.

Following sub-analysis of these 11 patients, mean CMT at 12 months was 350.3µm (SD 93.3µm), corresponding to a change in CMT of -139.5µm (SD 65.8µm) or 28.1% (12.9 %) between baseline and 12 months. Mean macular volume at 12 months was 8.5mm^3 (SD 0.8), corresponding to a change in macular volume of -0.6mm^3 (SD 0.6) between baseline and 12 months. Mean CMT at 6 months was similar at 360.7µm (SD 85.2µm), corresponding to a change in CMT of -129.1µm (SD 125.1µm) or 22.9% (29.7 %) (See figure 1) between baseline and 6 months. Mean macular volume at 6 months was 8.5mm^3 (SD 0.6) corresponding to a change in macular volume of -0.6mm^3 (SD 0.6) between baseline and 6 months. Figure 2 demonstrates SDOCT images of 2 responders taken at baseline and at 1 month post-baseline (after having received only a single ivA).

Mean ETDRS BCVA at 6 months was 67.5 letters (SD 10.1) corresponding to a gain of 3.8 letters (SD 6.8). Mean ETDRS BCVA at 12 months was 68.4 letters (SD 11.8) corresponding to a gain of 4.7 letters (SD 9.5) (Figure 3). It should be noted that 3 of 11 (27.3%) responders were graded as having disruption of the

ellipsoid zone within 1mm of the fovea on their baseline OCT scan. No improvement of vision was found in all 3 of these patients. Four of 11 (36.4%) responders were graded as having questionable presence of ERM within 3mm of the fovea.

Mean retinal sensitivity at 6 months was 4.93dB (SD 4.06), corresponding to a change in retinal sensitivity of -0.92dB (SD 2.03) between baseline and 6 months. Mean retinal sensitivity at 12 months was 4.48dB (SD 3.83), corresponding to a change in retinal sensitivity of -0.97dB (SD 1.92) between baseline and 12 months.

The median number of injections given in responders was 7 (IQR 6 to 10).

Additional data of non-responders

Eighteen out of 29 (62.1%) patients were classified as non-responders. Genetic mutations were identified and confirmed in 10 of 18 (55.6%) non-responders, which included: NRL (1 patient), RHO (1 patient), PRPF31 (1 patient), PRPF8 (1 patient), SNRNP200 (1 patient), USH2A (2 patients), TULP1 (1 patient), RP1 (1 patient) and IFT140 (1 patient). Six of 18 (33.3%) non-responders were graded as having disruption of the ellipsoid zone within 1mm of the fovea on their baseline OCT scan.
