# Supplementary material for: Prospective exploratory study to assess the safety and efficacy of aflibercept in cystoid macular oedema associated with retinitis pigmentosa
Source: Br J Ophthalmol. 2020 Sep 1;104(9):1203–8. doi: 10.1136/bjophthalmol-2019-315152 (PMC7577098; doi:10.1136/bjophthalmol-2019-315152)
Supplement: Supplementary data [file bjophthalmol-2019-315152s017.pdf]

Supplementary table 7: Ocular and Non-Ocular adverse events (AEs) and serious adverse events (SAEs) – 0-6 months after baseline

Study ID	Adverse Event	Start Date	Stop Date	Severity	Relationship to Study Treatment	Action Taken with Study Treatment	Outcome of AE	Expected	Serious
1	Floater in RE	11/08/2016	05/09/2016	Mild	Probably	None	Resolved	Yes	No
2	Sub-conjunctival haemorrhage	04/04/2016	05/04/2016	Mild	Not Related	None	Resolved	Yes	No
2	Blurring of vision	25/08/2016	06/09/2016	Mild	Possibly	None	Resolved	No	No
3	Contacted by pt to say similar blurring to 1st injection	07/06/2016	15/06/2016	Mild	Definitely	None	Resolved	Yes	No
3	Corneal epithelium dystrophy post injection	11/04/2016	12/04/2016	Mild	Definitely	None	Resolved	Yes	No
3	Back pain after bending down	10/10/2016	14/10/2016	Mild	Not Related	None	Resolved	No	No
4	Dry cornea	12/08/2016	14/08/2016	Mild	Definitely	None	Resolved	Yes	No
4	LUL lesion (chalazion)	19/10/2016		Mild	Not Related	None	AE ongoing	No	No
4	Grittiness / Dry eye	09/07/2016	16/07/2016	Mild	Probably	None	Resolved	Yes	No
5	Headache post - IVT	26/07/2016	26/07/2016	Mild	Probably	None	Resolved	Yes	No
5	Feeling lethargic	25/09/2016	30/11/2016	Mild	Unlikely	None	AE ongoing	No	No
5	Raised IOP post - IVT	03/05/2016	03/05/2016	Mild	Definitely	None	Resolved	Yes	No
5	Sub-conjunctival haemorrhage (5 days post IVT)	05/06/2016	15/06/2016	Mild	Definitely	None	Resolved	Yes	No
5	Tinnitus	10/07/2016		Mild	Not Related	None	AE ongoing	No	No
8	Dry ocular surface + pain	10/11/2016	16/11/2016	Moderate	Definitely	Discontinued permanently	Resolved	Yes	No
9	Headache	24/06/2016	26/06/2016	Mild	Not Related	None	Resolved	Yes	No
10	Sub-conjunctival haemorrhage	16/05/2016	19/05/2016	Mild	Definitely	None	Resolved	Yes	No
10	Sub-conjunctival haemorrhage	22/08/2016	26/08/2016	Mild	Definitely	None	Resolved	Yes	No
13	Bad back	15/08/2016	19/08/2016	Mild	Unlikely	None	Resolved	No	No
14	Viral cold with headache	18/10/2016	27/10/2016	Mild	Not Related	None	Resolved	No	No
14	Corneal abrasion	24/06/2016	25/06/2016	Mild	Definitely	None	Resolved	Yes	No
15	Grittiness/Blurring using laptop	01/10/2016	18/10/2016	Mild	Unlikely	None	Resolved	No	No
16	Conversion of prostate biopsy from benign to low-grade neoplasia	20/09/2016		Mild	Unlikely	None	AE ongoing	No	No
16	Vision is not as sharp	04/07/2016	06/12/2016	Mild	Possibly	None	AE ongoing	No	No

Study ID	Adverse Event	Start Date	Stop Date	Severity	Relationship to Study Treatment	Action Taken with Study Treatment	Outcome of AE	Expected	Serious
17	Bad back	14/11/2016	01/01/2017	Mild	Not Related	None	Resolved	Yes	No
17	Yag capsulotomy (on non-study eye LE)	28/10/2016	28/10/2016	Mild	Not Related	None	Resolved	Yes	No
17	Feeling Low	01/12/2016	01/05/2017	Mild	Not Related	None	Resolved	Yes	No
18	Viral Cold	22/09/2016	30/09/2016	Mild	Not Related	None	Resolved	No	No
19	Grittiness both eyes	12/09/2016	13/09/2016	Mild	Unlikely	None	Resolved	Yes	No
20	Labyrinthitis	02/08/2016	01/04/2017	Mild	Unlikely	None	Resolved	No	No
21	Sub-conjunctival haemorrhage	01/07/2016	03/07/2016	Mild	Definitely	None	Resolved	Yes	No
21	Soreness of eye	05/10/2016	07/10/2016	Mild	Definitely	None	Resolved	Yes	No
22	UTI	24/10/2016	28/10/2016	Mild	Not Related	None	Resolved	No	No
22	Dry corneal surface	02/11/2016	03/11/2016	Mild	Definitely	None	Resolved	Yes	No
22	Viral Illness	28/12/2016	22/01/2017	Mild	Not Related	None	Resolved	Yes	No
22	Sub-conjunctival haemorrhage	04/07/2016	09/07/2016	Mild	Definitely	None	Resolved	Yes	No
22	Dry corneal surface	04/07/2016	05/07/2016	Mild	Definitely	None	Resolved	Yes	No
22	Dry Eye	05/10/2016	30/10/2016	Mild	Definitely	None	Resolved	Yes	No
23	Bitten by mosquitto	10/08/2016	17/08/2016	Mild	Not Related	None	Resolved	No	No
24	Flare up of mental health issues	15/09/2016		Moderate	Unlikely	Discontinued permanently	AE ongoing	Yes	No
25	Heartburn	31/08/2016	25/09/2016	Mild	Unlikely	None	Resolved	No	No
25	Sub-conjunctival haemorrhage	06/12/2016	24/12/2016	Mild	Definitely	None	Resolved	Yes	No
26	Viral cold (nasal congestion)	19/11/2016	24/12/2016	Mild	Not Related	Delayed Dose	Resolved	Yes	No
27	Exacerbation of mental health illness	04/11/2016	17/11/2016	Mild	Unlikely	None	Resolved	No	No
27	Viral Illness	02/11/2016	09/11/2016	Mild	Not Related	None	Resolved	Yes	No
27	Punched in non-study eye (LE) with bruise under eye	27/08/2016	31/08/2016	Mild	Not Related	None	Resolved	No	No
28	Sub-conjunctival haemorrhage	05/10/2016	13/10/2016	Mild	Definitely	None	Resolved	Yes	No
