# Supplementary material for: Prospective exploratory study to assess the safety and efficacy of aflibercept in cystoid macular oedema associated with retinitis pigmentosa
Source: Br J Ophthalmol. 2020 Sep 1;104(9):1203–8. doi: 10.1136/bjophthalmol-2019-315152 (PMC7577098; doi:10.1136/bjophthalmol-2019-315152)
Supplement: Supplementary data [file bjophthalmol-2019-315152s018.pdf]

Supplementary table 8: Ocular and Non-Ocular Adverse Events (AEs) and Serious Adverse Events (SAEs) – 6- 12 months after baseline

Study ID	Adverse Event	Start Date	Stop Date	Severity	Relationship to Study Treatment	Action Taken with Study Treatment	Outcome of AE	Expected	Serious
4	Perforated Ear Drum	26/02/2017	03/03/2017	Mild	Not Related	None	Resolved	No	No
4	Ear Infection	26/02/2017	03/03/2017	Mild	Not Related	None	Resolved	No	No
4	Ear Infection	03/04/2017	09/04/2017	Mild	Not Related	None	Resolved	No	No
4	Viral cold	03/12/2016	05/12/2016	Mild	Not Related	None	Resolved	No	No
4	Vision not as clear	15/11/2016	06/12/2016	Mild	Definitely	None	Resolved	Yes	No
11	Subconjunctival haemorrhage	14/12/2016	17/12/2016	Mild	Definitely	None	Resolved	Yes	No
15	Low Mood	20/05/2017		Mild	Not Related	None	AE ongoing	No	No
15	Viral cold	13/12/2016	18/12/2016	Mild	Not Related	None	Resolved	Yes	No
16	Floater Right Eye	20/04/2017	27/04/2017	Mild	Definitely	None	Resolved	Yes	No
16	Grittiness after injection	06/02/2017	12/02/2017	Mild	Definitely	None	Resolved	Yes	No
16	Dry ocular surface	19/04/2017	20/04/2017	Mild	Definitely	None	Resolved	Yes	No
17	Viral Gastric Bug	09/04/2017	11/04/2017	Mild	Not Related	None	Resolved	No	No
17	Reduced central vision due to progression of underlying disease	12/05/2017		Moderate	Unlikely	Discontinued permanently	AE ongoing	Yes	No
21	Posterior vitreous detachment	17/03/2017		Mild	Possibly	None	AE ongoing	Yes	No
21	Anxiety	03/03/2017		Mild	Not Related	None	AE ongoing	No	No
21	Corneal abrasion + dry cornea	13/01/2017		Mild	Definitely	None	AE ongoing	Yes	No
27	Punched in the face just below RE (no sequelae)	20/03/2017	20/03/2017	Mild	Not Related	None	Resolved	No	No
27	Viral illness	21/01/2017	31/01/2017	Mild	Not Related	None	Resolved	Yes	No
28	Posterior vitreous detachment	16/02/2017		Mild	Possibly	None	AE ongoing	Yes	No
28	Vitreous floater	05/06/2017		Mild	Definitely	None	AE ongoing	Yes	No
30	Feeling low	17/02/2017	28/02/2017	Mild	Not Related	None	Resolved	Yes	No
30	Right eye posterior sub-capsular cataract (non study eye)	17/02/2017		Mild	Not Related	None	AE ongoing	Yes	No
