# Supplementary material for: Prospective exploratory study to assess the safety and efficacy of aflibercept in cystoid macular oedema associated with retinitis pigmentosa
Source: Br J Ophthalmol. 2020 Sep 1;104(9):1203–8. doi: 10.1136/bjophthalmol-2019-315152 (PMC7577098; doi:10.1136/bjophthalmol-2019-315152)
Supplement: Supplementary data [file bjophthalmol-2019-315152s019.pdf]

Supplementary Table 9: Ocular and Non-Ocular Adverse Events (AEs) and Serious Adverse Events (SAEs) – More than 12 months after baseline

Study ID	Adverse Event	Start Date	Stop Date	Severity	Relationship to Study Treatment	Action Taken with Study Treatment	Outcome of AE	Expected	Serious
11	Feeling tired from fasting	05/06/2017		Mild	Not Related	None	AE ongoing	No	No
15	Vitreous Floaters	16/06/2017		Mild	Possibly	None	AE ongoing	Yes	No
17	Reduced central vision	25/10/2017	12/05/2017	Mild	Possibly	Discontinued permanently	Resolved	No	Yes
27	Exacerbation of mental health illness	17/11/2017	23/11/2017	Mild	Unlikely	None	Resolved	No	No
30	Viral illness	05/12/2017	12/12/2017	Mild	Not Related	Delayed Dose	Resolved	Yes	No
