# Supplementary material for: Prospective exploratory study to assess the safety and efficacy of aflibercept in cystoid macular oedema associated with retinitis pigmentosa
Source: Br J Ophthalmol. 2020 Sep 1;104(9):1203–8. doi: 10.1136/bjophthalmol-2019-315152 (PMC7577098; doi:10.1136/bjophthalmol-2019-315152)
Supplement: Supplementary data [file bjophthalmol-2019-315152s020.pdf]

Safety: analysis of all study participants

Ocular AEs: Ocular AEs were the expected standard range of AEs seen with intravitreal injections (see supplementary tables 7 to 9). There were no cases of endophthalmitis or retinal detachment.

Ocular SAE: One participant reported sub-acute reduction of vision at week 32 despite being a ‘responder’, with testing demonstrating a reduction in vision of 14 ETDRS letters. Injections were immediately discontinued. Further assessments were undertaken including SDOCT, FAF, microperimetry and OCT-angiography (OCT-A). There was no demonstrable change in outer retinal lamination compared to baseline, with also no change in microperimetry or FAF compared to baseline, and no abnormality detected on OCT-A. The non-study eye had a baseline vision of 30 ETDRS letters due to advanced photoreceptor loss and it was therefore concluded that the reduction in vision was most likely secondary to progression of underlying RP rather than as a consequence of ivA. The patient remained in the study and attended the 6 and 12 month follow-up appointments.

Non-ocular/systemic AEs: All non-ocular/systemic AEs were reported during the study whether or not they were considered to be secondary to aflibercept. Non-ocular/systemic AEs included: back pain, headache, lethargy, tinnitus, viral cold, conversion of prostate biopsy from benign to low-grade neoplasia, feeling low/low mood, labyrinthitis, urine tract infection, relapse of mental illness, heartburn, perforated ear drum, ear infection, viral gastric illness, anxiety and mosquito bite.

Whilst the participant who developed labyrinthitis during the study was reassured that it was unlikely to be secondary to aflibercept, they decided that they would prefer to discontinue receiving injections. This patient remained in the study and attended the 6 and 12 month follow-up appointments.
